# Concerning Propaganda

**Published:** 1920-10-02

**Authors:** 


### CONCERNING PROPAGANDA.

The holiday season is over and done, and there is an accumulation of work, arrears in this storm-tossed world. Let us be up and doing.

I want to say something concerning propaganda. The more I think about it, the more convinced I feel that it is little understood in quarters where it is vitally essential. I may be regarded as propaganda mad when I suggest that every deserving public cause which lacks support owes its poverty to inefficient propaganda. This is either a delusion, or else it is a scientific fact of first-class importance, and cannot be ignored by the voluntary worker any more than the cause of disease can be ignored by the physician. If it is a delusion, then we are faced with an alternative from which most people will shrink. The alternative is that the nation must be branded as dishonest, void of a sense of justice, conscienceless.

### A New of Lost Art.

Have you seriously considered propaganda? It is by no means a new art, nor is it a lost art. Seven or eight centuries ago, before the days of printing and steam travelling, not to mention cinema shows and airships, when Europe was covered with forests, and when Englishmen fought their battles with bows and arrows, Peter the Hermit was able to rouse Europe to fight for possession of the Holy Sepulchre. Inspired by his trumpet-call, tens of thousands of men-at-arms of all nationalities united in the mighty enterprise of driving out the hosts of Islam. Who and what was this great propagandist? He was of the stature and ungainliness of a dwarf, emaciated by the austerities of his self-imposed discipline, head and feet bare mounted on an ass. But his soul was on fire, and the vehemence of his righteous indignation swept away all difficulties. In our own day we have witnessed highly organised propaganda suitable to a more complex and congested civilisation. For forty years before the war the highest intellects of Germany were engaged in political propaganda. The savagery and ruthlessness which the enemy displayed, and which shocked all the rest of humanity, do not indicate that there was any inherent rottenness in the men and women of Germany, but merely that the doctrine of hate was inculcated by royal command from kindergarten days to manhood. Even the national toasts and family greetings were carefully designed propaganda. Our own Red Cross was a masterpiece of propaganda; and, when the history of the war comes to be written, it will be found that a not unimportant factor in the victory of the Allies was British State propaganda in enemy countries.

While thus contemplating, I have in mind two appealing causes—the Nurse and the Hospital. Both are in sorry plight, and yet both are not alone popular national institutions, but, together, form an integral part of our civic economy. London is the world's money market, but when the Londoner proudly shows the visitor his monuments, foremost among them are the hospitals. They are in no wise inferior to the banks. The nurse is the soul of the hospital. Her uniform commands respect alike from rich and poor, in the palace and in the slum. It is vested, indeed, with a certain sacredness, for it is the mantle of Florence Nightingale. With her fortune, the country's grateful tribute, she founded and established the Order of Trained Nurses, whose members are to be found in every country in the world, savage or civilised.

### A Fault Within.

Am I to be told that hospitals are bankrupt and nurses sweated because British people are false to their ideals? The charge has only to be made in order to be scouted. The fault is to be found within rather than without. Hospital and nursing authorities fail to realise that propaganda is an art, and can be done only by artists. Governors of some of the biggest institutions have treated this as unskilled labour. They expended thousands of pounds in printing and publishing dry-as-dust statistics, which nobody read, and they expected the matron or the clerk to send round the fiery cross. A matron told me the other day that she had sent out some two or three hundred letters asking for funds. It never seemed to have occurred to this lady that it was not her job. I have seen many appeals about nurses' salaries, and I honestly affirm that they were so weak, so apologetic, so shamefaced, that I wondered, and I still wonder, if they were seriously intended. There was certainly nothing of Peter the Hermit about them. The same applies to the hospitals. Now and again there are sporadic efforts, with fine results. But, in truth, victory balls, charity concerts, sweepstakes, and tombolas are mediæval methods, of which the public are sick unto death. It is poor, weak, amateurish, dilletante propaganda, and the result is in proportion.

And, all the time, the British people are more ready than ever they were, to respond to the call of suffering humanity, and to do so handsomely.

IERNE.

